# Supplementary material for: Direct Antiviral Activity of IFN-Stimulated Genes Is Responsible for Resistance to Paramyxoviruses in ISG15-Deficient Cells
Source: J Immunol. 2020 May 18;205(1):261–71. doi: 10.4049/jimmunol.1901472 (PMC7311202; doi:10.4049/jimmunol.1901472)
Supplement: Data Supplement [file JI_1901472.zip › JI_1901472_Supplemental_Figure_1.pdf]

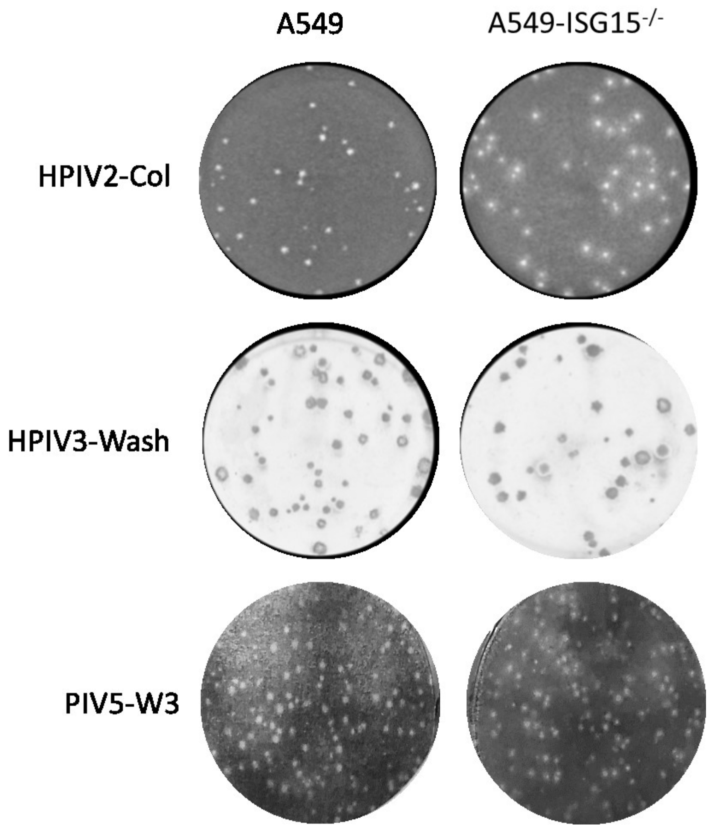

Supplementary Figure 1. No viral resistance in naïve ISG15-deficient cells. Near confluent A549 or A549-ISG15$^{-/-}$ (B8) cells in 6-well plates were infected with the indicated virus at dilution that allow the formation of discrete plaques. Following 6 days infection, cells were fixed and either stained with toluidine blue O (HPIV2 strain Collindale and PIV5 strain W3-infected cells) or immunostained (HPIV3 strain Washington using antibodies specific for HPIV3 nucleoprotein).
